# Propensity-Score Matched Analysis of the Effectiveness of Baricitinib in Patients With Coronavirus Disease 2019 (COVID-19) Using Nationwide Real-World Data: An Observational Matched Cohort Study From the Japan COVID-19 Task Force

**DOI:** 10.1093/ofid/ofad311

**Published:** 2023-06-08

**Authors:** Hiromu Tanaka, Shotaro Chubachi, Ho Namkoong, Yasunori Sato, Takanori Asakura, Ho Lee, Shuhei Azekawa, Shiro Otake, Kensuke Nakagawara, Takahiro Fukushima, Mayuko Watase, Kaori Sakurai, Tatsuya Kusumoto, Yasushi Kondo, Katsunori Masaki, Hirofumi Kamata, Makoto Ishii, Yuko Kaneko, Naoki Hasegawa, Soichiro Ueda, Mamoru Sasaki, Takehiro Izumo, Minoru Inomata, Naoki Miyazawa, Yasuhiro Kimura, Yusuke Suzuki, Norihiro Harada, Masako Ichikawa, Tohru Takata, Hiroyasu Ishikura, Takashi Yoshiyama, Hiroyuki Kokuto, Koji Murakami, Hirohito Sano, Tetsuya Ueda, Naota Kuwahara, Akiko Fujiwara, Takashi Ogura, Takashi Inoue, Takahiro Asami, Yoshikazu Mutoh, Ichiro Nakachi, Rie Baba, Koichi Nishi, Mayuko Tani, Junko Kagyo, Mizuha Hashiguchi, Tsuyoshi Oguma, Koichiro Asano, Masanori Nishikawa, Hiroki Watanabe, Yukinori Okada, Ryuji Koike, Yuko Kitagawa, Akinori Kimura, Seiya Imoto, Satoru Miyano, Seishi Ogawa, Takanori Kanai, Koichi Fukunaga

**Affiliations:** Division of Pulmonary Medicine, Department of Internal Medicine, Keio University School of Medicine, Tokyo, Japan; Division of Pulmonary Medicine, Department of Internal Medicine, Keio University School of Medicine, Tokyo, Japan; Department of Infectious Diseases, Keio University School of Medicine, Tokyo, Japan; Department of Preventive Medicine and Public Health, Keio University School of Medicine, Tokyo, Japan; Division of Pulmonary Medicine, Department of Internal Medicine, Keio University School of Medicine, Tokyo, Japan; Division of Pulmonary Medicine, Department of Internal Medicine, Keio University School of Medicine, Tokyo, Japan; Division of Pulmonary Medicine, Department of Internal Medicine, Keio University School of Medicine, Tokyo, Japan; Division of Pulmonary Medicine, Department of Internal Medicine, Keio University School of Medicine, Tokyo, Japan; Division of Pulmonary Medicine, Department of Internal Medicine, Keio University School of Medicine, Tokyo, Japan; Division of Pulmonary Medicine, Department of Internal Medicine, Keio University School of Medicine, Tokyo, Japan; Division of Pulmonary Medicine, Department of Internal Medicine, Keio University School of Medicine, Tokyo, Japan; Division of Pulmonary Medicine, Department of Internal Medicine, Keio University School of Medicine, Tokyo, Japan; Division of Pulmonary Medicine, Department of Internal Medicine, Keio University School of Medicine, Tokyo, Japan; Division of Rheumatology, Department of Internal Medicine, Keio University School of Medicine, Tokyo, Japan; Division of Pulmonary Medicine, Department of Internal Medicine, Keio University School of Medicine, Tokyo, Japan; Division of Pulmonary Medicine, Department of Internal Medicine, Keio University School of Medicine, Tokyo, Japan; Division of Pulmonary Medicine, Department of Internal Medicine, Keio University School of Medicine, Tokyo, Japan; Division of Rheumatology, Department of Internal Medicine, Keio University School of Medicine, Tokyo, Japan; Department of Infectious Diseases, Keio University School of Medicine, Tokyo, Japan; JCHO (Japan Community Health Care Organization) Saitama Medical Center, Internal Medicine, Saitama, Japan; JCHO (Japan Community Health Care Organization) Saitama Medical Center, Internal Medicine, Saitama, Japan; Japanese Red Cross Medical Center, Tokyo, Japan; Japanese Red Cross Medical Center, Tokyo, Japan; Department of Respiratory Medicine, Saiseikai Yokohamashi Nanbu Hospital, Yokohama, Japan; Department of Respiratory Medicine, Saiseikai Yokohamashi Nanbu Hospital, Yokohama, Japan; Department of Respiratory Medicine, Kitasato University Kitasato Institute Hospital, Tokyo, Japan; Department of Respiratory Medicine, Juntendo University Faculty of Medicine and Graduate School of Medicine, Tokyo, Japan; Department of Respiratory Medicine, Juntendo University Faculty of Medicine and Graduate School of Medicine, Tokyo, Japan; Department of Infection Control, Fukuoka University Hospital, Fukuoka, Japan; Department of Emergency and Critical Care Medicine, Fukuoka University Faculty of Medicine, Fukuoka, Japan; Fukujuji Hospital, Kiyose, Japan; Fukujuji Hospital, Kiyose, Japan; Department of Respiratory Medicine, Tohoku University Graduate School of Medicine, Sendai, Japan; Department of Respiratory Medicine, Tohoku University Graduate School of Medicine, Sendai, Japan; Department of Respiratory Medicine, Osaka Saiseikai Nakatsu Hospital, Osaka, Japan; Internal Medicine, Internal Medicine Center, Showa University Koto Toyosu Hospital, Tokyo, Japan; Internal Medicine, Internal Medicine Center, Showa University Koto Toyosu Hospital, Tokyo, Japan; Kanagawa Cardiovascular and Respiratory Center, Yokohama, Japan; Internal Medicine, Sano Kosei General Hospital, Sano, Japan; Internal Medicine, Sano Kosei General Hospital, Sano, Japan; Department of Infectious Diseases, Tosei General Hospital, Seto, Japan; Saiseikai Utsunomiya Hospital, Utsunomiya, Japan; Department of Infectious Diseases, Tosei General Hospital, Seto, Japan; Ishikawa Prefectural Central Hospital, Kanazawa, Japan; Ishikawa Prefectural Central Hospital, Kanazawa, Japan; Keiyu Hospital, Yokohama, Japan; Keiyu Hospital, Yokohama, Japan; Division of Pulmonary Medicine, Department of Medicine, Tokai University School of Medicine, Isehara, Japan; Division of Pulmonary Medicine, Department of Medicine, Tokai University School of Medicine, Isehara, Japan; Department of Respiratory Medicine, Fujisawa City Hospital, Fujisawa, Japan; Department of Respiratory Medicine, Fujisawa City Hospital, Fujisawa, Japan; Department of Statistical Genetics, Osaka University Graduate School of Medicine, Suita, Japan; Department of Genome Informatics, Graduate School of Medicine, the University of Tokyo, Tokyo, Japan; Laboratory for Systems Genetics, RIKEN Center for Integrative Medical Sciences, Kanagawa, Japan; Medical Innovation Promotion Center, Tokyo Medical and Dental University, Tokyo, Japan; Department of Surgery, Keio University School of Medicine, Tokyo, Japan; Institute of Research, Tokyo Medical and Dental University, Tokyo, Japan; Division of Health Medical Intelligence, Human Genome Center, the Institute of Medical Science, the University of Tokyo, Tokyo, Japan; M&D Data Science Center, Tokyo Medical and Dental University, Tokyo, Japan; Department of Pathology and Tumor Biology, Kyoto University, Kyoto, Japan; Division of Gastroenterology and Hepatology, Department of Internal Medicine, Keio University School of Medicine, Tokyo, Japan; Division of Pulmonary Medicine, Department of Internal Medicine, Keio University School of Medicine, Tokyo, Japan

**Keywords:** baricitinib, COVID-19, invasive mechanical ventilation, real-world data

## Abstract

**Background:**

To determine the effectiveness of baricitinib in patients with coronavirus disease 2019 (COVID-19), investigate whether baricitinib prevents the need for invasive mechanical ventilation and identify patient subgroups that would benefit from baricitinib.

**Methods:**

This observational matched-cohort study was conducted by the Japan COVID-19 Task Force, a nationwide multicenter consortium. Patients with COVID-19 aged ≥18 years were identified from 70 hospitals in Japan. Among patients with confirmed COVID-19 from February 2020 to September 2021, those receiving baricitinib were propensity-score matched with controls.

**Results:**

Among 3309 patients, 144 propensity score-matched pairs were identified. Thirteen (9.0%) patients in the baricitinib group and 27 (18.8%) in the control group required invasive mechanical ventilation during the disease course (odds ratio, 0.43). Although the baricitinib group had more severe disease, there were no significant differences in the intensive care unit admission rates (odds ratio, 1.16) and mortality rates (odds ratio, 0.74) between groups. In subgroup analyses, baricitinib was associated with a significant reduction in the need for invasive mechanical ventilation in patients requiring oxygen support (odds ratio, 0.28), with rapid shadow spread on chest radiography (odds ratio, 0.11), or treated with remdesivir (odds ratio, 0.27), systemic corticosteroids (odds ratio, 0.31), or anticoagulants (odds ratio, 0.17).

**Conclusions:**

Baricitinib is effective at preventing the need for invasive mechanical ventilation in patients with COVID-19.

## Teaser

Real-world data from patients in 70 hospitals in Japan showed that baricitinib is effective at preventing the need for invasive mechanical ventilation in patients with COVID-19.

Coronavirus disease 2019 (COVID-19) can be fatal, causing severe pneumonia and a cytokine storm [1], and has become a social crisis with many deaths worldwide. This pandemic has continued even after the widespread administration of vaccines, and it is essential to develop treatment methods for patients with severe disease to prepare for future surges in the national epidemic.

A characteristic feature of treatment for severe COVID-19 is the use of drugs against the host immune response combined with antiviral therapy [2]. The effectiveness of systemic corticosteroids for inflammation-induced lung injury has been widely recognized in the RECOVERY clinical trial [3]. In addition, other drugs targeting many proinflammatory cytokines and their signaling pathways have been widely studied internationally. Recently, 2 randomized control trials in patients hospitalized with COVID-19 showed the efficacy of baricitinib (BARI), a Janus kinase 1/2 inhibitor discovered by artificial intelligence [4]. The ACTT-2 trial demonstrated that BARI combined with remdesivir treatment reduced the recovery time, especially among patients with high-flow oxygen or noninvasive ventilation [5]. The COV-BARRIER trial reported that BARI treatment resulted in lower 28-day mortality than the standard treatment, without an increase in venous thromboembolism [6]. Therefore, BARI combined with remdesivir treatment for hospitalized patients with COVID-19 requiring oxygen support was approved by the US Food and Drug Administration in November 2020, followed by Japan in April 2021. Therefore, BARI is widely recognized as an anti-inflammatory treatment option in combination with systemic corticosteroids for patients with severe COVID-19.

The Japan COVID-19 Task Force was established as a nationwide multicenter consortium in Japan to overcome COVID-19 by collecting and analyzing clinical information and specimens from patients with COVID-19 in more than 100 institutions nationwide since February 2020. The Task Force has been working to elucidate the genetic characteristics of the Japanese population affected by COVID-19 [7] and reported their clinical characteristics, including the low mortality rate of Japanese patients [8]. To our knowledge, only a few large-scale studies have compared mortality in patients with COVID-19 treated with BARI versus other treatments using a real-world database, with a recent single-center report from Japan [9]. This study aimed to determine the effectiveness of BARI in preventing invasive mechanical ventilation (IMV) support and to identify patients who would benefit from BARI treatment using a real-world nationwide COVID-19 database.

## METHODS

### Study Design and Settings

**Figure 1.**
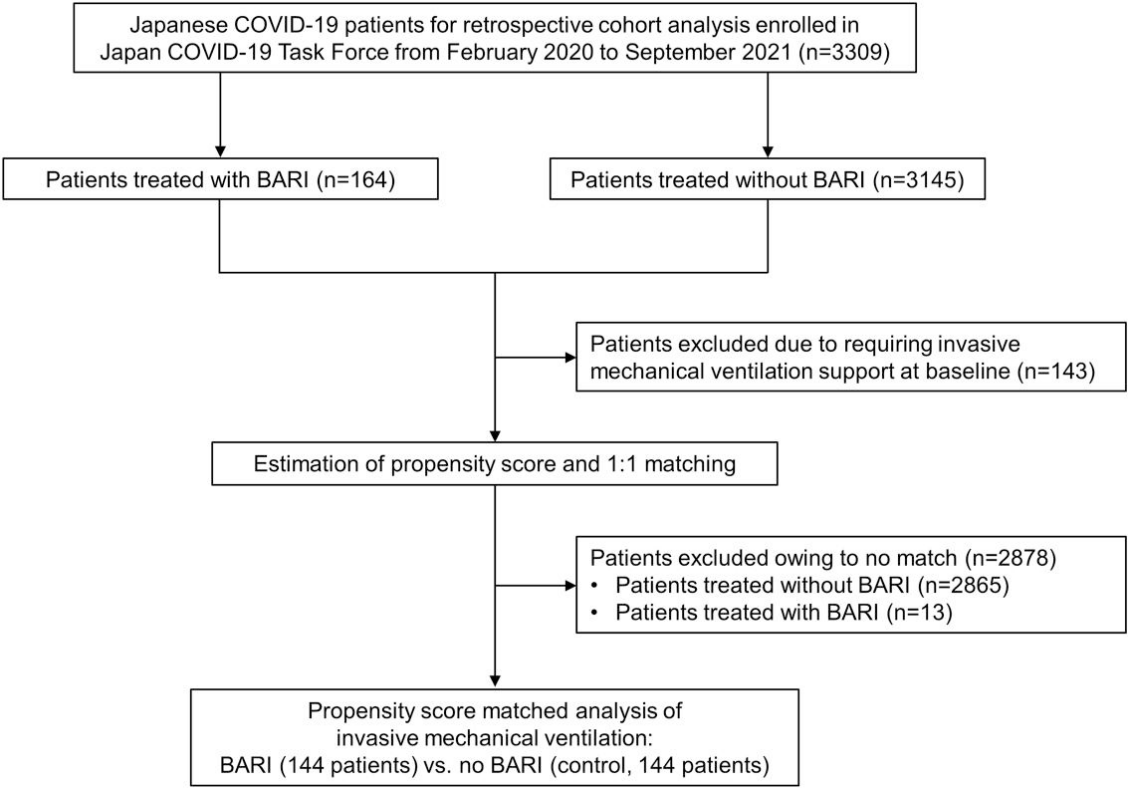
Flowchart of patients included in the analysis. BARI, baricitinib; COVID-19, coronavirus disease 2019.

This observational matched-cohort study used a prospectively collected database. The Japan COVID-19 Task Force collected clinical information on patients with COVID-19 from 70 hospitals nationwide in Japan [7, 8]. All patients in this study provided informed consent, and the study was approved by the ethics committee of the Keio University School of Medicine (20200061) and related research institutions. A Consolidated Standards of Reporting Trials flow diagram of the patient selection process is shown in Figure 1. In total, 3309 Japanese patients with COVID-19 aged ≥18 years who were enrolled between February 2020 and September 2021 were selected for analysis.

We initially classified the overall analysis population into 2 groups: those treated with and without BARI, and we evaluated the clinical characteristics of each group. For patients treated with BARI, we assessed its specific use, outcomes, and frequency of adverse events. The clinical characteristics of the BARI-treated and non-BARI-treated groups were compared using propensity score matching (PSM). The primary outcome was set as the requirement for IMV support during the disease course because the mortality rate and intensive care unit (ICU) admission rate have already been reported to be low in the Japanese database [8], and secondary outcomes included mortality rate, ICU admission rate, and worst disease severity. To clarify the population in which treatment with BARI had a favorable effect, a subgroup analysis was performed, and the odds ratio (OR) for requiring IMV support was calculated in each subgroup. As previously reported [8, 10], the worst disease severity was established as follows: critical - patients requiring support using high-flow oxygen devices, IMV, extracorporeal membrane oxygenation, or death; severe - patients requiring support using low-flow oxygen devices; mild - symptomatic patients not requiring oxygen support; and asymptomatic - asymptomatic patients not requiring oxygen support.

### Data Collection

The following clinical information was collected: demographics, comorbidities, clinical symptoms and signs, laboratory test data, radiographic imaging, oxygen support, treatment, and outcome. For patients using BARI, information on drug use was also collected, including dosing, duration of administration, and adverse effects. Symptoms and signs included those reported on referral or admission and those observed during hospitalization. Laboratory and radiographic data were collected within 48 hours of the initial visit or admission. The rapid spread of the shadow on chest radiography was defined as the enlargement of findings to more than 50% of the entire lung field within 48 hours of referral. Treatment included medications for COVID-19, such as favipiravir, remdesivir, anticoagulant, tocilizumab, and systemic corticosteroids. Missing or absent patient background data were noted as unknown.

### Statistical Analysis

Continuous variables were compared using the unpaired *t* test, and categorical variables were compared using the χ^2^ test. *P* values were 2-tailed, and *P* < .05 was considered statistically significant. The propensity score was calculated using a logistic regression model including age [11], sex [11], body mass index [12], oxygen support at baseline, medical history (hypertension [13], diabetes mellitus [14], hyperuricemia [15], cardiovascular disease [16], malignancy [17], autoimmune disease [18], chronic obstructive pulmonary disease [19], asthma, chronic liver disease, and chronic kidney disease) [20], and duration from onset to hospitalization, some of which are known risk factors for severe COVID-19. Propensity-matched cohorts of the BARI group and control group (standard therapy without BARI) were derived and compared using a 1:1 ratio with greedy matching on the propensity score, with a caliper of 0.2 standard deviations of the propensity score logit with no replacement. We examined standardized differences and variance ratios to determine whether the matched cohort had balanced patient characteristics. Logistic regression was performed to estimate ORs with 95% confidence intervals (CIs) for the analysis of outcomes and subgroups after matching. All statistical analyses were conducted using the JMP 16 program and SAS version 9.4 (SAS Institute, Inc., Cary, NC). Visualization was performed using GraphPad Prism 8 (GraphPad Software, San Diego, CA) and the R package *ggalluvial*.

## RESULTS

### Domestic Epidemic Waves of Coronavirus Disease 2019 in Japan and the Number of Included Patients Treated With Bari

Supplementary Figure 1 shows the number of patients treated with BARI in this study and the number of new patients with COVID-19 and the number of cases of critical disease in Japan. Japan experienced 5 domestic epidemic waves in the past (April and August 2020 and January, May, and August 2021), with the fifth wave in 2021 showing an outstanding number of new patients. Data from 164 patients treated with BARI were collected after December 2020, and most of the patients were from the fourth and fifth waves after approval of the domestic indication.

### Status, Outcome, and Adverse Effects of Treatment With Bari

Supplementary Table 1 shows the specific use, frequency of outcomes, and adverse events in patients treated with BARI. The median duration from disease onset to BARI administration was 8 days (95% CI, 6–10 days). Mortality within 28 days of BARI administration was limited to 2.4%. Of the 22 patients on IMV support, 20 patients (90.9%) were extubated, 1 patient could not be extubated, and 1 patient died 5 days after extubation. Of the patients, 86.6% progressed from requiring oxygen support to not requiring it within a median of 10 days (95% CI, 7–14 days) after BARI administration. The incidence of bacterial pneumonia and bacteremia complications was 3.0% and 1.8%, respectively. We observed only 1 patient developing a small thrombosis in the jugular vein, but no patients developed venous thromboembolism or pulmonary embolism after the administration of BARI. As for liver dysfunction, 13 of 17 patients already had elevated liver function at baseline.

### Comparison of Clinical Characteristics of Patients With Coronavirus Disease 2019 Treated With and Without Bari

Table 1 shows the clinical characteristics of 164 patients treated with BARI and 3145 patients treated without BARI before and after PSM. The worst disease severity in the entire population was 17.5% for critical and 31.3% for severe, with an IMV support rate of 12.4%, ICU admission rate of 20.8%, and mortality rate of 3.4%. In the BARI group, 97 patients (59.5%) required oxygen support at baseline, which was significantly higher than in the control group (OR, 4.13; 95% CI, 2.99–5.69). The 2 groups differed in several baseline characteristics; however, the balance was satisfactory after matching.

**Table 1.** Clinical Characteristics of Japanese COVID-19 Patients by Treatment Group

	Propensity Score-Matched Population						Unmatched Population					
Parameters	BARI Treatment Group (n = 144)		Control Treatment Group (n = 144)		*P* Value	SMD	BARI Treatment Group (n = 164)		Control Treatment Group (n = 3145)		*P* Value	SMD
	No. of Patients With Data	n (%) or Median (IQR)	No. of Patients With Data	n (%) or Median (IQR)			No. of Patients with Data	n (%) or Median (IQR)	No. of Patients with Data	n (%) or Median (IQR)		
Oxygen support at baseline^a^	144		144		.937	0.043	163		3130		<.001	0.742
Not require oxygen support		60 (41.7)		58 (40.3)				66 (40.5)		2308 (73.7)		
Require low-flow oxygen support		79 (54.9)		80 (55.6)				85 (52.1)		664 (21.2)		
Require high-flow oxygen support		5 (3.5)		6 (4.2)				5 (3.1)		22 (0.7)		
Require IMV or ECMO		0 (0.0)		0 (0.0)				7 (4.3)		136 (4.4)		
Age^a^	144	54 (48–64)	144	54 (44–65)	.412	0.097	164	54 (48–64)	3145	58 (45–71)	.191	0.118
Sex^a^	144		144		.763	0.036	164		3145		<.001	0.324
Male		116 (80.6)		118 (81.9)				132 (80.5)		2087 (66.4)		
Female		28 (19.4)		26 (18.1)				32 (19.5)		1058 (33.6)		
BMI^a^	144	25.7 (22.7–28.6)	144	25.2 (22.4–28.9)	.747	0.038	162	25.8 (22.7–28.7)	2909	24.0 (21.6–27.1)	<.001	0.278
Smoking history	135		136		.363	0.111	155		2892		.650	0.037
Never		67 (49.6)		60 (44.1)				79 (51.0)		1528 (52.8)		
Previously or currently		68 (50.4)		76 (55.9)				76 (49.0)		1364 (47.2)		
Medical history^a^												
Hypertension	144	48 (33.3)	144	40 (27.8)	.306	0.121	164	55 (33.5)	3104	1071 (34.5)	.800	0.020
Diabetes mellitus	144	35 (24.3)	144	32 (22.2)	.676	0.049	162	42 (25.9)	3109	655 (21.1)	.141	0.115
Cardiovascular disease	144	13 (9.0)	144	10 (6.9)	.514	0.077	164	14 (8.5)	3112	321 (10.3)	.464	0.061
Malignancy	144	8 (5.6)	144	8 (5.6)	1.000	<0.001	164	10 (6.1)	3093	213 (6.9)	.697	0.032
Autoimmune disease	144	3 (2.1)	144	3 (2.1)	1.000	<0.001	164	3 (1.8)	3100	126 (4.1)	.152	0.132
COPD	144	5 (3.5)	144	3 (2.1)	.473	0.085	164	6 (3.7)	3094	128 (4.1)	.764	0.025
Asthma	144	5 (3.5)	144	8 (5.6)	.395	0.100	160	8 (5.0)	3068	223 (7.3)	.278	0.095
Hyperuricemia	144	12 (8.3)	144	18 (12.5)	.247	0.137	163	14 (8.6)	3099	319 (10.3)	.484	0.058
Chronic liver disease	144	4 (2.8)	144	2 (1.4)	.409	0.097	161	5 (3.1)	3039	133 (4.4)	.439	0.067
Chronic kidney disease	144	5 (3.5)	144	5 (3.5)	1.000	<0.001	161	7 (4.4)	3016	221 (7.3)	.154	0.127
Hospitalization												
Onset to hospitalization^a^ (days)	135	7 (5–9)	135	7 (5–10)	.694	0.118	163	7 (5–9)	2942	5 (3–8)	<.001	0.330
Symptoms												
Fever (≥37.5°C)	144	136 (94.4)	140	121 (86.4)	.021	0.275	164	153 (93.3)	3106	2494 (80.3)	<.001	0.391
Cough	143	126 (88.1)	141	94 (66.7)	<.001	0.530	163	138 (84.7)	3076	1869 (60.8)	<.001	0.557
Sputum	141	63 (44.7)	139	33 (23.7)	<.001	0.453	161	70 (43.5)	3056	843 (27.6)	<.001	0.337
Sore throat	143	39 (27.3)	138	30 (21.7)	.281	0.129	162	40 (24.7)	3047	750 (24.6)	.982	0.002
Rhinorrhea	142	15 (10.6)	139	16 (11.5)	.800	0.030	162	15 (9.3)	3054	431 (14.1)	.082	0.152
Dysgeusia	143	34 (23.8)	140	25 (17.9)	.220	0.146	162	37 (22.8)	3051	564 (18.5)	.166	0.108
Dysosmia	143	23 (16.1)	140	18 (12.9)	.441	0.092	163	24 (14.7)	3052	492 (16.1)	.636	0.039
Shortness of breath	144	98 (68.1)	141	67 (47.5)	<.001	0.425	163	110 (67.5)	3041	1112 (36.6)	<.001	0.651
Abdominal pain	144	11 (7.6)	138	7 (5.1)	.378	0.105	164	12 (7.3)	3055	101 (3.3)	.007	0.180
Diarrhea	143	29 (20.3)	137	19 (13.9)	.155	0.171	163	31 (19.0)	3048	531 (17.4)	.601	0.041
Nausea	142	25 (17.6)	138	13 (9.4)	.046	0.241	162	27 (16.7)	3022	265 (8.7)	<.001	0.240
Fatigue	142	118 (83.1)	140	74 (52.9)	<.001	0.685	162	131 (80.9)	3067	1645 (53.6)	<.001	0.606
Laboratory tests												
WBC (/μL)	141	5300 (4000–6680)	144	5550 (3920–7100)	.618	0.059	161	5400 (4000–6800)	3059	5100 (4000–6800)	.823	0.019
Neutrophil (/μL)	140	4090 (2890–5410)	140	3990 (2690–5630)	.733	0.041	159	4130 (2880–5660)	2974	3510 (2520–5060)	.140	0.128
Lymphocyte (/μL)	140	830 (610–1090)	140	880 (630–1210)	.188	0.158	159	840 (620–1110)	2980	980 (700–1340)	<.001	0.410
Albumin (g/dL)	142	3.4 (3.1–3.8)	139	3.6 (3.1–4.1)	.016	0.288	162	3.4 (3.1–3.8)	3017	3.8 (3.3–4.2)	<.001	0.523
AST (IU/L)	142	45 (33–60)	144	40 (25–59)	.503	0.079	162	45 (33–59)	3078	31 (23–47)	.018	0.220
ALT (IU/L)	142	32 (23–51)	144	35 (20–62)	.145	0.173	162	32 (24–54)	3084	26 (17–45)	.452	0.076
LDH (IU/L)	142	380 (283–466)	144	294 (211–411)	<.001	0.468	162	380 (280–466)	3077	248 (194–342)	<.001	0.640
Creatinine (mg/dL)	142	0.88 (0.75–1.04)	144	0.86 (0.71–1.07)	.980	0.003	162	0.87 (0.74–1.04)	3083	0.83 (0.67–1.01)	.264	0.120
Ferritin (ng/mL)	138	783 (433–1221)	117	571 (320–1081)	.179	0.170	158	783 (447–1286)	2531	395 (184–750)	<.001	0.497
KL-6 (U/mL)	125	296 (206–414)	106	278 (216–405)	.742	0.043	145	301 (213–426)	2240	243 (180–357)	.034	0.174
PCT (ng/mL)	113	0.10 (0.06–0.23)	113	0.07 (0.05–0.12)	.819	0.030	130	0.10 (0.06–0.23)	2305	0.06 (0.04–0.12)	.931	0.010
CRP (mg/dL)	142	7.38 (4.31–10.55)	143	4.37 (1.44–9.41)	.004	0.342	162	7.27 (4.11–11.11)	3068	3.00 (0.68–7.34)	<.001	0.554
D-dimer (μg/mL)	142	1.0 (0.8–1.4)	135	1.0 (0.6–1.4)	.141	0.175	162	1.0 (0.8–1.4)	2939	0.9 (0.5–1.4)	.216	0.136
Chest radiography findings												
GGO bilateral/unilateral	144	122 (84.7)/10 (6.9)	143	103 (72.0)/11 (7.7)	.012	0.353	164	136 (82.9)/14 (8.5)	2980	1631 (54.7)/351 (11.8)	<.001	0.687
Consolidation bilateral/unilateral	144	63 (43.8)/25 (17.4)	144	48 (33.3)/12 (8.3)	.002	0.421	164	71 (43.3)/27 (16.5)	2957	640 (21.6)/270 (9.1)	<.001	0.609
Rapid spread of the shadow^b^	133	62 (46.6)	133	14 (10.5)	<.001	0.871	150	64 (42.7)	2741	242 (8.8)	<.001	0.839
Chest CT findings												
GGO bilateral/unilateral	132	119 (90.2)/7 (5.3)	141	118 (83.7)/9 (6.4)	.206	0.218	151	136 (90.1)/8 (5.3)	2800	2042 (72.9)/271 (9.7)	<.001	0.470
Consolidation bilateral/unilateral	132	73 (55.3)/6 (4.6)	141	64 (45.4)/11 (7.8)	.203	0.218	151	84 (55.6)/6 (4.0)	2755	932 (33.8)/229 (8.3)	<.001	0.458
Complication after referral												
Bacterial infection	143	9 (6.3)	143	16 (11.2)	.143	0.174	163	13 (8.0)	3096	336 (10.9)	.247	0.099
Heart failure	142	0 (0.0)	142	3 (2.1)	.082	0.208	162	0 (0.0)	3084	65 (2.1)	.062	0.208
Thromboembolism	141	0 (0.0)	144	2 (1.4)	.160	0.168	160	0 (0.0)	3056	99 (3.2)	.021	0.259
Kidney dysfunction^c^	141		140		.026	0.327	160		2985		.004	0.246
Moderate		34 (24.1)		22 (15.7)				36 (22.5)		393 (13.2)		
Severe		6 (4.3)		1 (0.7)				6 (3.8)		115 (3.9)		
Treatment for COVID-19												
Antibiotics	143	27 (18.9)	143	46 (32.2)	.010	0.308	163	36 (22.1)	3110	763 (24.5)	.478	0.058
Antiviral drug												
Favipiravir	143	6 (4.2)	143	37 (25.9)	<.001	0.637	163	6 (3.7)	3113	850 (27.3)	<.001	0.691
Remdesivir	144	142 (98.6)	142	69 (48.6)	<.001	1.378	164	162 (98.8)	3104	1024 (33.0)	<.001	1.928
Anticoagulant	143	94 (65.7)	143	58 (40.6)	<.001	0.521	163	107 (65.6)	3110	881 (28.3)	<.001	0.806
Tocilizumab	143	31 (21.7)	143	29 (20.3)	.772	0.034	163	33 (20.3)	3099	297 (9.6)	<.001	0.303
Systemic corticosteroid	144	132 (91.7)	144	99 (68.8)	<.001	0.601	164	152 (92.7)	3130	1575 (50.3)	<.001	1.063
Primary outcome												
IMV support	144	13 (9.0)	144	27 (18.8)	.017	0.284	164	22 (13.4)	3145	389 (12.4)	.692	0.031
Secondary outcome												
ICU admission	143	39 (27.3)	143	35 (24.5)	.589	0.064	163	49 (30.1)	3112	633 (20.3)	.003	0.225
Death discharge	144	3 (2.1)	144	4 (2.8)	.702	0.045	164	5 (3.0)	3139	106 (3.4)	.820	0.019
Worst disease severity	144		144		<.001	0.747	164		3145		<.001	1.274
Asymptomatic		0 (0.0)		1 (0.7)				0 (0.0)		94 (3.0)		
Mild		7 (4.9)		45 (31.3)				8 (4.9)		1594 (50.7)		
Severe		89 (61.8)		64 (44.4)				95 (57.9)		940 (29.9)		
Critical		48 (33.3)		34 (23.6)				61 (37.2)		517 (16.4)		

Abbreviations: ALT, alanine transaminase; AST, aspartate aminotransferase; BARI, baricitinib; BMI, body mass index; COPD, chronic obstructive pulmonary disease; COVID-19, coronavirus disease 2019; CRP, C-reactive protein; CT, computed tomography; ECMO, extracorporeal membrane; GGO, ground-glass opacity; ICU, intensive care unit; IMV, invasive mechanical ventilation; IQR, interquartile range; KL-6, Krebs von den Lungen-6; LDH, lactate dehydrogenase; PCT, procalcitonin; SMD, standardized mean difference; WBC, white blood cell.

NOTE: Data are presented as N (%) or median (interquartile range).

a The propensity score was calculated using a logistic regression model including these variables.

b Defined as enlargement of chest radiography findings to more than 50% of the entire lung field within 48 hours of referral.

c Moderate and severe kidney dysfunction are defined as estimated glomerular filtration rate <60 and <30 mL/min/1.73 m^2^, respectively.

**Figure 2.**
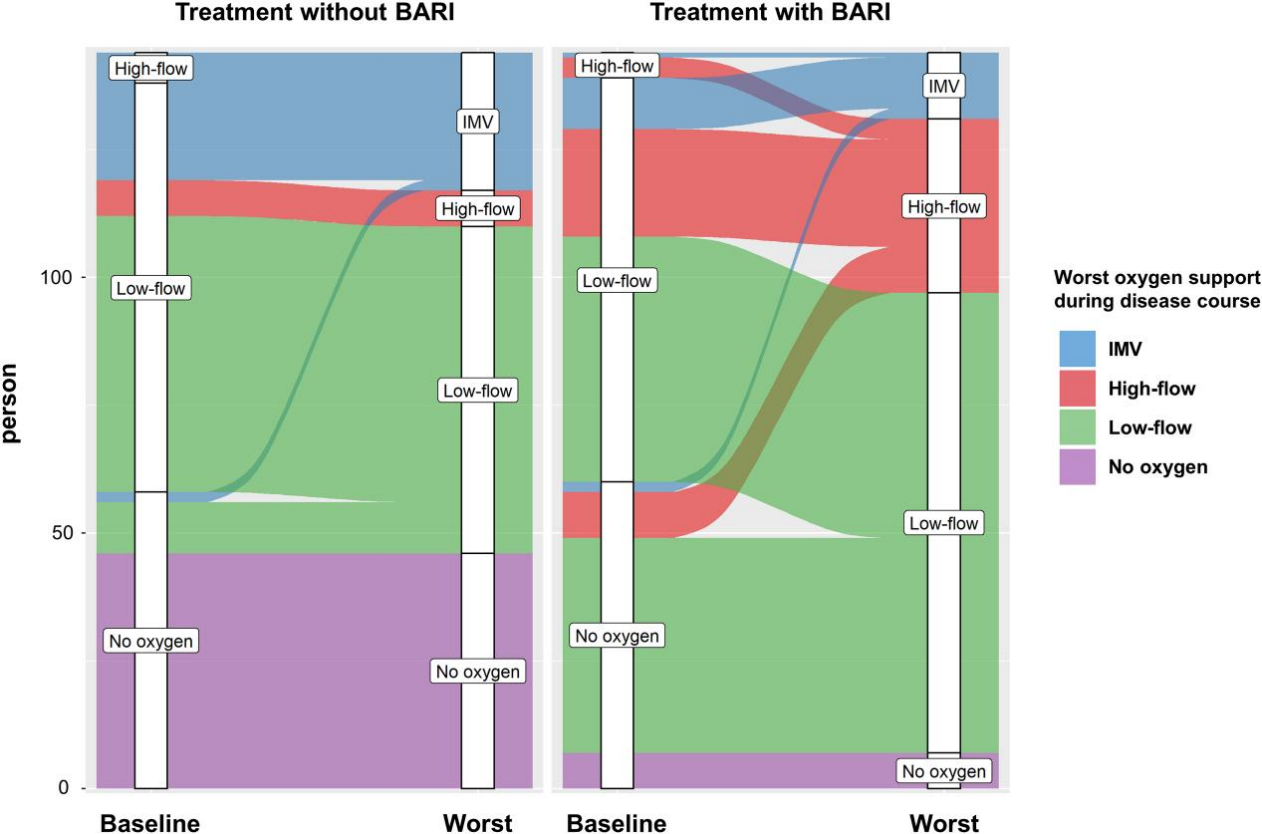
Changes in the requirement for oxygen support during the disease course in the propensity-matched cohort. The number of patients requiring oxygen support at baseline and at their worst during hospitalization: invasive mechanical ventilation (IMV), high-flow oxygen, low-flow oxygen, and no requirement of oxygen support. Although the requirement for oxygen support at baseline was matched, the baricitinib (BARI) group showed lower rates of IMV induction than the control group.

Figure 2 shows the changes in the requirement for oxygen support from baseline to worst during the disease course in the propensity-matched cohort. The proportion of patients requiring oxygen support during the course of the disease was significantly higher in the BARI group (137 patients, 95.1%) than in the control group (98 patients, 68.0%). Regarding the primary outcome, 13 patients (9.0%) in the BARI group and 27 patients (18.8%) in the control group (OR, 0.43; 95% CI .21–.87) required IMV support after matching (Table 1). Regarding the secondary outcomes, there were no significant differences in the ICU admission rate (OR, 1.16; 95% CI, .68–1.97) and mortality rate (OR, 0.74; 95% CI, .16–3.39), although the worst disease severity was more severe in the BARI group. Before matching, the BARI-treated patients showed more systemic and respiratory symptoms, such as fever (93.3%), fatigue (80.9%), cough (84.7%), and shortness of breath (67.5%), and some inflammatory markers, such as lactate dehydrogenase, ferritin, Krebs von den Lungen-6, and C-reactive protein, were significantly higher than those in the control group. However, some clinical characteristics were not significantly different between the 2 groups after PSM. On imaging tests, there were still significantly more cases of bilateral ground-glass opacity (OR, 2.15; 95% CI, 1.20–3.86) and rapid deterioration (OR, 7.42; 95% CI, 3.87–14.2) in the BARI group after matching. In the BARI group, more patients were administered remdesivir (98.6%), systemic corticosteroids (91.7%), and anticoagulants (65.7%).

### Comparison of Clinical Characteristics Between Populations Requiring and Not Requiring Invasive Mechanical Ventilation Support in Each Treatment Group

Invasive mechanical ventilation-supported patients showed higher Krebs von den Lungen-6 and C-reactive protein levels in both treatment groups (Supplementary Table 2). We also observed a higher frequency of rapid spread of the shadow (OR, 5.26; 95% CI, 1.66–16.7) on chest radiographs in IMV-supported patients in the control treatment group, whereas there were no significant differences in the BARI group. Regarding other treatments, the frequency of remdesivir, systemic corticosteroids, and anticoagulant use was higher in IMV-supported patients only in the control treatment group.

### Subgroup Analysis of the Odds Ratio for the Primary Outcome

**Figure 3.**
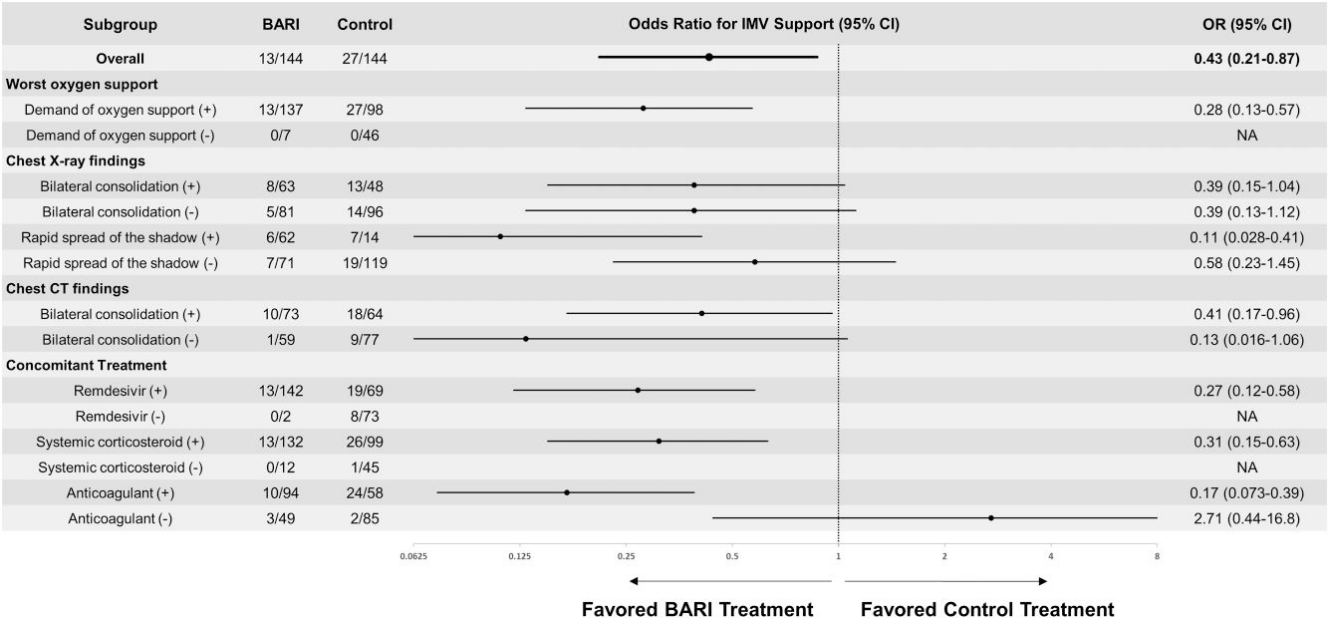
Subgroup analysis of odds ratios for requiring invasive mechanical ventilation (IMV) based on the propensity-matched patient sample. Odds ratio (OR) >1.0, favored non-baricitinib (BARI) treatment (control); OR <1.0, favored BARI treatment. CI, confidence interval; CT, computed tomography; NA; not available.

Figure 3 shows the evaluation of the OR for the primary outcome, requiring IMV support, in each subgroup based on the propensity score-matched population (OR > 1.0, favored non-BARI [control] treatment; OR < 1.0, favored BARI treatment). The subgroup of developing a demand for oxygen support during the disease course showed a low OR of 0.28 (95% CI, .13–0.57). For the imaging variables, the subgroup with rapid spread of the shadow on chest radiography showed an OR of 0.11 (95% CI, .028–.41). As for concomitant medications, the subgroups with the following 3 drugs all showed low ORs: remdesivir, OR = 0.27 (95% CI, .12–.58); systemic corticosteroids, OR = 0.31 (95% CI, .15–.63); and anticoagulant, OR = 0.17 (95% CI, .073–.39).

## DISCUSSION

Our study showed the effectiveness of BARI treatment using the largest real-world dataset, mostly in patients requiring oxygen support, including during the course. Baricitinib was approved in Japan in April 2021, and, as shown in Figure 1, most of the patients treated with BARI were extracted from the fourth and fifth waves of the domestic epidemic wave. The mortality rate was 3.4% in the control group and 3.0% in the BARI group, which was not significantly different from that reported in the Japanese database [21]. We observed that BARI was administered to more patients who required oxygen support and had a rapid spread of shadows on chest radiography. The COVID-19 disease course is divided into 2 stages: an early viral stage and an immune response stage that leads to increased inflammation and a cytokine storm, which is associated with dyspnea [1, 22]. In this study, we confirmed that the median duration from onset to BARI administration was 8 days, indicating that BARI has an anti-inflammatory effect in the latter stage. Similar to previous studies in Japan [23], the frequency of adverse events with BARI observed in this study was low, suggesting no apparent safety concerns.

In this study, we observed that the IMV support rate was significantly lower in the BARI group, despite more patients in the BARI group requiring oxygen support during the disease course, and confirmed that BARI clearly contributed to intubation avoidance. Compared with ACTT-2 [5] and COV-BARRIER [6], there were more patients without oxygen support at baseline, especially a smaller proportion of patients requiring high-flow oxygen support. Conversely, in this study, the BARI group showed higher levels of lactate dehydrogenase, C-reactive protein, ferritin, and Krebs von den Lungen-6 than the control group before PSM. Because these 4 inflammatory parameters indicating severe disease were consistent with the inclusion criteria in COV-BARRIER, it is likely that BARI was administered to more patients with severe disease in the clinical setting. We evaluated the characteristics of the BARI group based on PSM after adjusting for oxygen demand at baseline. As shown in Figure 2, the BARI group showed a significantly lower IMV support rate than the control group, although more patients required oxygen support during the disease course. We also observed that the ICU admission rate remained equivalent in the BARI and control groups; however, we believe that ICU admission rates do not reflect the disease severity of COVID-19. One reason for this was that many institutions admitted patients to ICU, even in mild cases, for infection control purposes. Another reason is that patients supported by high-flow oxygen devices are, in principle, admitted to ICU in Japan. In the subgroup analysis, the effect of BARI in avoiding IMV support was remarkable in the population requiring oxygen support during the disease course, which is consistent with the highly effective BARI population observed in ACTT-2 and COV-BARRIER. Although the ACTT-2 subanalysis did not clarify the effectiveness of BARI in Asians, these favorable results indicate the effectiveness of BARI in Japanese patients with COVID-19 with oxygen demand, even considering the difference in the rate of concomitant systemic corticosteroids treatment.

Because BARI was effective in avoiding IMV support in patients requiring oxygen support during the disease course, we evaluated the characteristics of the patient groups that should be actively treated with BARI. The subgroup analysis revealed the expected patients and those with the rapid spread of the shadow observed on chest radiography. We believe that BARI is effective in cases of progressive pneumonia because Janus kinase inhibitors nonselectively inhibit many cytokines. Patients with severe COVID-19 present elevated levels of many cytokines associated with the Janus kinase/signal transducer and activator of the transcription pathway [24], which has been shown to be associated with the demand for oxygen support [25]. The results of this study support the possibility of identifying a population in which early BARI administration prevents more critical conditions simply by evaluating chest radiographs in the clinical setting. Our subgroup analysis also suggested that BARI treatment in combination with remdesivir, systemic corticosteroids, and anticoagulants may be beneficial, consistent with the COV-BARRIER and ACTT-2 studies. The combination of BARI with corticosteroids has a favorable anti-inflammatory effect in cases of severe disease that have already entered the immune response phase, as shown in previous studies [26, 27]. Thrombosis prophylaxis, including concomitant use of anticoagulants, is a principle when BARI is administered, and anticoagulants were used in almost all patients in the ACTT2 trial but in only 65.6% of the BARI-treated patients in this study. Although the benefit of therapeutic heparin for COVID-19 has been partially demonstrated [28, 29], this study is the first to show the benefit of combining heparin with BARI, which may be related to crosstalk between the immune response and coagulation systems [30].

To eliminate potential bias, we applied PSM. First, we considered the selection bias in that the virulence of the infecting virus strains was not evaluated. As reported previously [31], different strains of viral variants have been observed in different epidemic waves with variations in disease severity. In the present study, the BARI group had the majority of the cases from April 2021 onward and therefore contained most of the alpha and delta strains, whereas the control group had fewer cases from this time period. In addition, we should be aware of the differences in comedications between groups, which was a major bias in this study. However, PSM allowed us to include patients in the control group who had background factors that would have supported treatment with first-line BARI treatment if the indications had been approved. In the future, this analysis is expected to be performed with more bias-free matching by increasing the target population. Second, the mortality rate of the study population was lower than that of other countries, and caution should be exercised when interpreting comparisons with randomized controlled trials. We considered the possibility that many inpatients in our study would have been treated as outpatients in other countries. However, the influence of the genetic background characteristics of the Japanese population should be considered, and the Japan COVID-19 Task Force is still in the process of genetic analysis. Third, although the present PSM matched the patient background at referral, the medications were not adjusted because they were intervention factors during the course of the study. Differences in the use of antibiotics, remdesivir, and systemic corticosteroids between both treatment groups may have overestimated the effectiveness of BARI treatment. With further case counts, we will evaluate the additive effectiveness of BARI on systemic corticosteroids in real-world data. Another limitation was that the number of patients in each subgroup was insufficient. We expect that our results will be supplemented by a multicenter prospective study to collect more patients treated with BARI.

## CONCLUSIONS

We analyzed real-world data of patients with COVID-19 and, together with subgroup analyses, succeeded in confirming the effectiveness and safety of BARI in patients requiring oxygen support during the disease course. Collecting reports on BARI administration, including this study, will lead to the standardization of treatment of severe COVID-19, and we believe that this effective treatment is expected to be a powerful solution to the global medical crisis.

## Supplementary Material

ofad311_Supplementary_Data

## Supplementary Data

Supplementary materials are available at *Open Forum Infectious Diseases* online. Consisting of data provided by the authors to benefit the reader, the posted materials are not copyedited and are the sole responsibility of the authors, so questions or comments should be addressed to the corresponding author.
