# Supplementary material for: Propensity-Score Matched Analysis of the Effectiveness of Baricitinib in Patients With Coronavirus Disease 2019 (COVID-19) Using Nationwide Real-World Data: An Observational Matched Cohort Study From the Japan COVID-19 Task Force
Source: Open Forum Infect Dis. 2023 Jun 8;10(7):ofad311. doi: 10.1093/ofid/ofad311 (PMC10334380; doi:10.1093/ofid/ofad311)
Supplement: ofad311_Supplementary_Data [file ofad311_supplementary_data.docx]

**Supplementary Information**

**Propensity-Score Matched Analysis of the Effectiveness of Baricitinib in Patients with COVID-19 Using Nationwide Real-World Data: An Observational Matched Cohort Study from The Japan COVID-19 Task Force**

Hiromu Tanaka, Shotaro Chubachi, Ho Namkoong, Yasunori Sato, Takanori Asakura, Ho Lee, Shuhei Azekawa, Shiro Otake, Kensuke Nakagawara, Takahiro Fukushima, Mayuko Watase, Kaori Sakurai, Tatsuya Kusumoto, Yasushi Kondo, Katsunori Masaki, Hirofumi Kamata, Makoto Ishii, Yuko Kaneko, Naoki Hasegawa, Soichiro Ueda, Mamoru Sasaki, Takehiro Izumo, Minoru Inomata, Naoki Miyazawa, Yasuhiro Kimura, Yusuke Suzuki, Norihiro Harada, Masako Ichikawa, Tohru Takata, Hiroyasu Ishikura, Takashi Yoshiyama, Hiroyuki Kokuto, Koji Murakami, Hirohito Sano, Tetsuya Ueda, Naota Kuwahara, Akiko Fujiwara, Takashi Ogura, Takashi Inoue, Takahiro Asami, Yoshikazu Mutoh, Ichiro Nakachi, Rie Baba, Koichi Nishi, Mayuko Tani, Junko Kagyo, Mizuha Hashiguchi, Tsuyoshi Oguma, Koichiro Asano, Masanori Nishikawa, Hiroki Watanabe, Yukinori Okada, Ryuji Koike, Yuko Kitagawa, Akinori Kimura, Seiya Imoto, Satoru Miyano, Seishi Ogawa, Takanori Kanai, Koichi Fukunaga


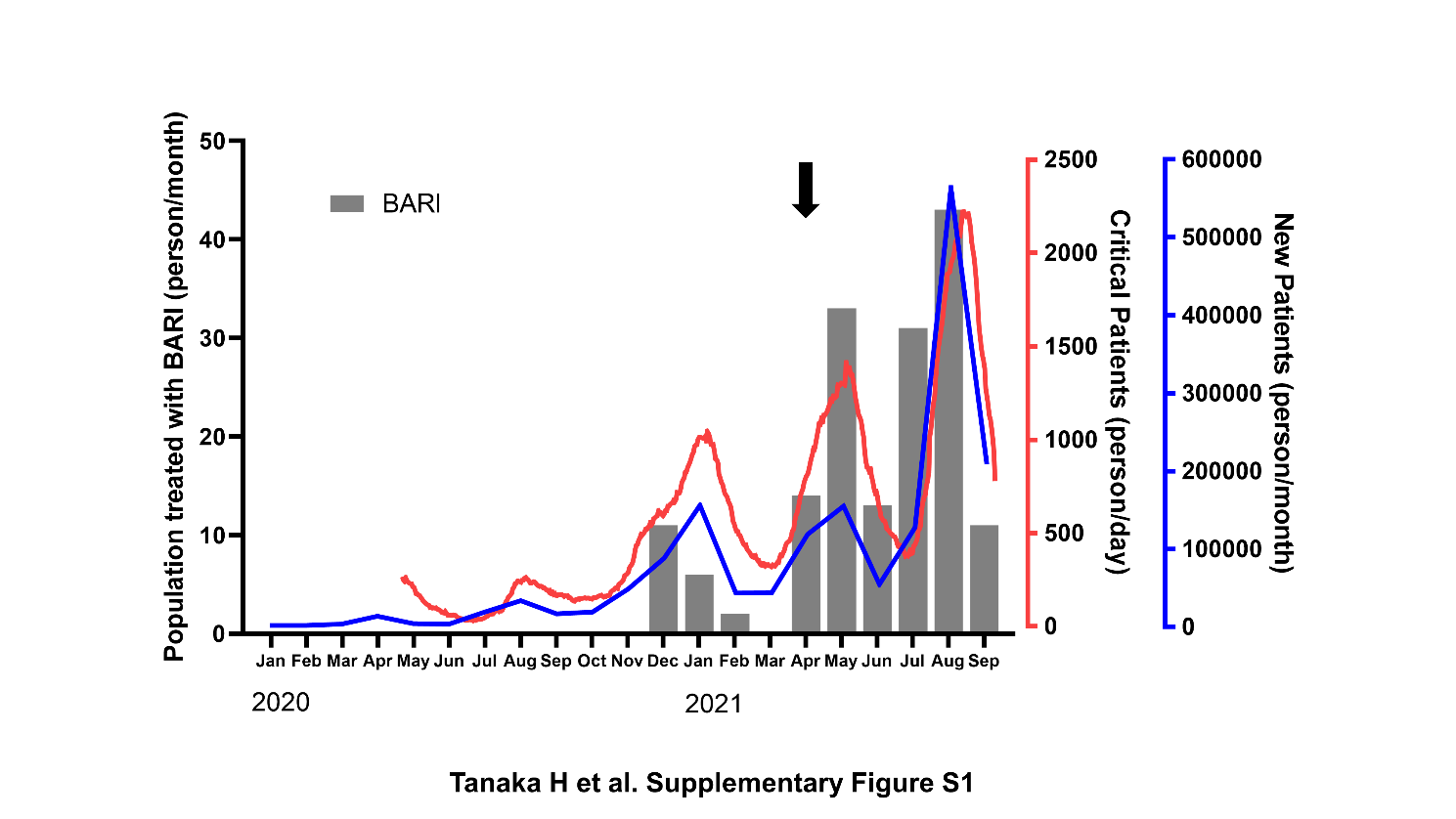


**Supplementary Figure 1**. Number of patients treated with baricitinib in this study (grey bar), and the number of new patients with COVID-19 (blue line) and cases of critical disease (red line) in Japan. Black arrow represents the date of April 23, 2021, when baricitinib treatment for COVID-19 was approved in Japan.

BARI, baricitinib; COVID-19, coronavirus disease 2019

| **Supplementary Table 1. Status, outcomes, and adverse events of baricitinib treatment** | | |
| --- | --- | --- |
| **Parameters** | **No. of patients with data** | **n (%) or median (IQR)** |
| Status of drug use |  |  |
| Dosing | 158 |  |
| maximum 2 mg |  | 15 (9.5) |
| maximum 4 mg |  | 143 (90.5) |
| Duration of administration, days | 164 | 9 (7-14) |
| From onset to administration, days | 163 | 8 (6-10) |
| Outcome |  |  |
| Death by day 28, n | 164 | 4 (2.4) |
| Successful extubation from IMV, n | 22 | 20 (90.9) |
| Duration of IMV, days | 21 | 8 (5-13) |
| Duration of hospitalization, days | 164 | 14 (11-20) |
| Duration of ICU stay, days | 40 | 7 (5-13) |
| Duration of supplementary oxygen therapy, days | 155 | 10 (7-14) |
| Adverse effect |  |  |
| Neutropenia (<600/μL) | 164 | 1 (0.6) |
| Lymphopenia (<500/μL) | 164 | 16 (9.8) |
| Anemia | 164 | 0 (0.0) |
| Dyslipidemia | 161 | 0 (0.0) |
| CK elevation | 163 | 0 (0.0) |
| Liver dysfunction | 164 | 17 (10.4) |
| Kidney dysfunction | 164 | 7 (4.3) |
| Venous thromboembolism | 164 | 0 (0.0) |
| Pulmonary embolism | 164 | 0 (0.0) |
| Interstitial pneumonitis | 164 | 2 (1.2) |
| Heart failure | 164 | 0 (0.0) |
| Reactivation of hepatitis B | 164 | 0 (0.0) |
| Herpes simplex | 164 | 0 (0.0) |
| Herpes zoster | 164 | 0 (0.0) |
| Urinary tract infection | 164 | 3 (1.8) |
| Bacterial pneumonia | 164 | 5 (3.0) |
| Bacteremia | 164 | 3 (1.8) |
| Critical hemorrhage | 164 | 2 (1.2) |
| Data are presented as N (%) or median (IQR). Abbreviations: BARI, baricitinib; CK, creatine kinase; ICU, intensive care unit; IMV, invasive mechanical ventilation; IQR, interquartile range | | |

| **Supplementary Table 2. Comparison of clinical characteristics of patients requiring and not requiring invasive mechanical ventilation** | | | | | |  |
| --- | --- | --- | --- | --- | --- | --- |
| **Parameters** | **BARI treatment group (n=144)** | | | **Control treatment group (n=144)** | | |
|  | **Require  IMV support (n=13)** | **Not require IMV support (n=131)** | ***P*-value** | **Require IMV support (n=27)** | **Not require IMV support (n=117)** | ***P*-value** |
| Smoking history |  |  | 0.122 |  |  | 0.502 |
| Never | 3 (27.3) | 64 (51.6) |  | 13 (50.0) | 47 (42.7) |  |
| Previously or currently | 8 (72.7) | 60 (48.4) |  | 13 (50.0) | 63 (57.3) |  |
| Symptoms |  |  |  |  |  |  |
| Fever (≥37.5ºC) | 12 (92.3) | 124 (94.7) | 0.724 | 22 (84.6) | 99 (86.8) | 0.765 |
| Cough | 12 (100.0) | 114 (87.0) | 0.184 | 19 (76.0) | 75 (64.7) | 0.275 |
| Sputum | 3 (25.0) | 60 (46.5) | 0.152 | 6 (25.0) | 27 (23.5) | 0.873 |
| Sore throat | 3 (25.0) | 36 (27.5) | 0.854 | 6 (26.1) | 24 (20.9) | 0.580 |
| Rhinorrhea | 0 (0.0) | 15 (11.5) | 0.213 | 2 (8.3) | 14 (12.2) | 0.592 |
| Dysgeusia | 0 (0.0) | 34 (26.0) | 0.043 | 1 (4.4) | 24 (20.5) | 0.064 |
| Dysosmia | 0 (0.0) | 23 (17.6) | 0.113 | 1 (4.4) | 17 (14.5) | 0.182 |
| Shortness of breath | 11 (84.6) | 87 (66.4) | 0.179 | 20 (80.0) | 47 (40.5) | <0.001 |
| Fatigue | 10 (83.3) | 108 (83.1) | 0.982 | 17 (70.8) | 57 (49.1) | 0.053 |
| Laboratory tests |  |  |  |  |  |  |
| WBC (/μL) | 8010 (5060-8950) | 5100 (3900-6480) | 0.004 | 7300 (4800-10100) | 5400 (3860-6750) | <0.001 |
| Neutrophil (/μL) | 6240 (3950-7900) | 3780 (2820-5230) | 0.003 | 6230 (4290-9250) | 3760 (2630-5030) | <0.001 |
| Lymphocyte (/μL) | 690 (480-1040) | 840 (610-1110) | 0.196 | 680 (450-910) | 910 (680-1290) | <0.001 |
| Albumin (g/L) | 3.1 (2.9-3.4) | 3.5 (3.1-3.8) | 0.021 | 2.9 (2.6-3.2) | 3.8 (3.3-4.3) | <0.001 |
| LDH (IU/L) | 415 (357-559) | 380 (281-461) | 0.169 | 437 (312-572) | 259 (202-393) | <0.001 |
| Creatinine (mg/dL) | 1.03 (0.82-1.25) | 0.86 (0.75-1.04) | 0.016 | 0.88 (0.71-1.09) | 0.84 (0.71-1.04) | 0.086 |
| Ferritin (ng/mL) | 1119 (414-1367) | 757 (443-1167) | 0.545 | 994 (683-1671) | 517 (272-996) | <0.001 |
| KL-6 (U/mL) | 420 (284-698) | 289 (201-391) | 0.002 | 357 (261-592) | 257 (202-377) | 0.048 |
| PCT (ng/mL) | 0.20 (0.09-0.34) | 0.09 (0.06-0.17) | 0.997 | 0.17 (0.05-0.44) | 0.06 (0.05-0.10) | 0.845 |
| CRP (mg/dL) | 11.47 (7.42-17.62) | 7.08 (4.00-10.19) | 0.002 | 10.95 (4.52-19.83) | 3.39 (0.92-8.05) | <0.001 |
| D-dimer (μg/mL) | 0.9 (0.5-1.6) | 1.0 (0.8-1.4) | 0.407 | 1.2 (0.9-2.4) | 0.9 (0.6-1.3) | 0.027 |
| Chest X-ray findings |  |  |  |  |  |  |
| GGO 　bilateral/unilateral | 12 (92.3) / 1 (7.7) | 110 (84.0) / 9 (6.9) | 0.522 | 23 (85.2) / 2 (7.4) | 80 (69.0) / 9 (7.8) | 0.172 |
| Consolidation 　bilateral/unilateral | 8 (61.5) / 3 (23.1) | 55 (42.0) / 22 (16.8) | 0.189 | 13 (48.2) / 2 (7.4) | 35 (29.9) / 10 (8.6) | 0.190 |
| Rapid spread of the shadow^*^ | 6 (46.2) | 56 (46.7) | 0.972 | 7 (26.9) | 7 (6.5) | 0.002 |
| Chest CT findings |  |  |  |  |  |  |
| GGO 　bilateral/unilateral | 11 (100.0) / 0 (0.0) | 108 (89.3) / 7 (5.8) | 0.519 | 27 (100.0) / 0 (0.0) | 91 (79.8) / 9 (7.9) | 0.039 |
| Consolidation 　bilateral/unilateral | 10 (90.9) / 0 (0.0) | 63 (52.1) / 6 (5.0) | 0.046 | 18 (66.7) / 1 (3.7) | 46 (40.4) / 10 (8.8) | 0.046 |
| Complication after referral |  |  |  |  |  |  |
| Bacterial infection | 3 (23.1) | 6 (4.6) | 0.009 | 10 (37.0) | 6 (5.2) | <0.001 |
| Heart failure | 0 (0.0) | 0 (0.0) |  | 2 (8.0) | 1 (0.9) | 0.024 |
| Thromboembolism | 0 (0.0) | 0 (0.0) |  | 2 (7.4) | 0 (0.0) | 0.003 |
| Kidney dysfunction^**^ |  |  | 0.010 |  |  | 0.473 |
| Moderate | 6 (46.2) | 28 (21.9) |  | 6 (23.1) | 16 (14.0) |  |
| Severe | 2 (15.4) | 4 (3.1) |  | 0 (0.0) | 1 (0.9) |  |
| Treatment for COVID-19 |  |  |  |  |  |  |
| Antibiotics | 9 (75.0) | 18 (13.7) | <0.001 | 21 (77.8) | 25 (21.6) | <0.001 |
| Antiviral drug |  |  |  |  |  |  |
| Favipiravir | 1 (8.3) | 5 (3.8) | 0.455 | 8 (29.6) | 29 (25.0) | 0.621 |
| Remdesivir | 13 (100.0) | 129 (98.5) | 0.654 | 19 (70.4) | 50 (43.5) | 0.012 |
| Anticoagulant | 10 (76.9) | 84 (64.6) | 0.373 | 24 (92.3) | 34 (29.1) | <0.001 |
| Tocilizumab | 6 (46.2) | 25 (19.2) | 0.025 | 15 (55.6) | 14 (12.1) | <0.001 |
| Systemic corticosteroid | 13 (100.0) | 119 (90.8) | 0.254 | 26 (96.3) | 73 (62.4) | <0.001 |
| Data are presented as N (%) or median (interquartile range). Abbreviations: BARI, baricitinib; COVID-19, coronavirus disease; CRP, C-reactive protein; CT, computed tomography; GGO, ground glass opacity; IMV, invasive mechanical ventilation; KL-6, Krebs von den Lungen-6; LDH, lactate dehydrogenase; PCT, procalcitonin; WBC, white blood cell ^*^Defined as enlargement of chest X-ray findings to more than 50% of the entire lung field within 48 hours of referral. ^**^Moderate and severe kidney dysfunction are defined as an estimated glomerular filtration rate <60 and <30 mL/min/1.73 m^2^, respectively. | | | | | | |

**Acknowledgement**

We would like to thank all participants involved in this study and all members of the Japan COVID-19 Task Force regularly engaged in clinical and research work on COVID-19. All members contributed to this study. The list of members is shown below.

**Chiba University**

Koutaro Yokote, Taka-Aki Nakada, Ryuzo Abe, Taku Oshima, Tadanaga Shimada

**Daini Osaka Police Hospital**

Kensuke Kanaoka, Shoichi Ihara, Kiyoshi Komuta

**Eiju General Hospital**

Fumitake Saito, Keiko Mitamura, Masao Hagihara, Junichi Ochi, Tomoyuki Uchida

**Fujioka General Hospital**

Mitsuru Motegi

**Fujisawa City Hospital**

Masanori Nishikawa, Makoto Masuda, Aya Wakabayashi, Hiroki Watanabe, Suguru Ueda

**Fukujuji Hospital**

Takashi Yoshiyama, Ken Ohta, Hiroyuki Kokuto, Hideo Ogata, Yoshiaki Tanaka, Kenichi Arakawa, Masafumi Shimoda, Takeshi Osawa

**Fukuoka Tokushukai Hospital**

Nobuhiro Kodama, Yasunari Kaneyama, Shunsuke Maeda, Takashige Kuraki, Takemasa Matsumoto

**Fukuoka University Hospital**

Tohru Takata, Yoshihiko Nakamura, Kota Hoshino, Junichi Maruyama, Hiroyasu Ishikura

**Fukushima Medical University**

Yoko Shibata, Yoshinori Tanino, Takefumi Nikaido, Hiroyuki Minemura, Yuki Sato

**Gifu University**

Yuichiro Kitagawa, Tetsuya Fukuta, Takahito Miyake, Shozo Yoshida, Shinji Ogura

**Gunma University**

Masakiyo Yatomi, Toshitaka Maeno

**The Institute of Medical Science, The University of Tokyo**

Takayoshi Hyugaji, Eigo Shimizu, Kotoe Katayama, Seiya Imoto

**International University of Health and Welfare Shioya Hospital**

Akira Umeda, Kazuya Miyagawa, Hisato Shimada, Mayu Endo, Yoshiyuki Ohira

**Ishikawa Prefectural Central Hospital**

Koichi Nishi, Masaru Nishitsuji, Mayuko Tani, Junya Suzuki, Hiroki Nakatsumi

**JA Toride Medical Hospital**

Shinichi Ogawa, Tomouki Ogata, Shoichiro Ishihara

**Japan Community Health Care Organization Kanazawa Hospital**

Kazuyoshi Watanabe

**Japan Community Health Care Organization Saitama Medical Center**

Soichiro Ueda, Mamoru Sasaki, Ai Tada, Masayoshi Miyawaki, Masaomi Yamamoto, Eriko Yoshida, Reina Hayashi, Tomoki Nagasaka, Sawako Arai, Yutaro Kaneko, Kana Sasaki

**Japanese Red Cross Medical Center**

Takehiro Izumo, Minoru Inomata, Naoyuki Kuse, Nobuyasu Awano, Mari Tone

**Juntendo University**

Norihiro Harada, Masako Ichikawa, Kazuhisa Takahashi, Toshio Naito, Makoto Hiki, Yasushi Matsushita, Haruhi Takagi, Ryousuke Aoki, Ai Nakamura, Sonoko Harada, Hitoshi Sasano

**Kanagawa Cardiovascular and Respiratory Center**

Takashi Ogura, Hideya Kitamura, Eri Hagiwara, Kota Murohashi, Hiroko Okabayashi

**Kansai Electric Power Hospital**

Yuichiro Yamada, Takuya Hashino, Masato Shinoki

**Kansai Medical University General Medical Center**

Fukuki Saito, Yasushi Nakamori, Kazuhisa Yoshiya, Tomoyuki Yoshihara, Daiki Wada, Hiromu Iwamura, Syuji Kanayama, Shuhei Maruyama

**Kanto Rosai Hospital**

Yoshihiro Hirai, Hidetoshi Kawashima, Atsuya Narita, Kazuki Niwa, Yoshiyuki Sekikawa

**Kawasaki Municipal Ida Hospital**

Yasushi Nakano, Yukiko Nakajima, Ryusuke Anan, Ryosuke Arai, Yuko Kurihara, Yuko Harada, Kazumi Nishio

**Keio University**

Ho Namkoong, Shotaro Chubachi, Hiromu Tanaka, Yasunori Sato, Takanori Asakura, Tatsuya Kusumoto, Kaori Sakurai, Mayuko Watase, Takahiro Fukushima, Ho Lee, Shiro Otake, Kensuke Nakagawara, Yasushi Kondo, Yuko Kaneko, Hiroshi Nishihara, Yohei Mikami, Rino Ishihara, Yuta Matsubara, Junko Hamamoto, Emmy Yanagita, Mari Katsumata, Atsuho Morita, Takunori Ogawa, Hiroki Kabata, Katsunori Masaki, Hirofumi Kamata, Shinnosuke Ikemura, Satoshi Okamori, Hideki Terai, Junichi Sasaki, Hiroshi Morisaki, Yoshifumi Uwamino, Kosaku Nanki, Sho Uchida, Shunsuke Uno, Tomoyasu Nishimura, Toshiro Sato, Makoto Ishii, Naoki Hasegawa, Yuko Kitagawa, Takanori Kanai, Koichi Fukunaga

**Keiyu Hospital**

Tetsuya Shiomi, Kazuma Yagi, Mizuha Hashiguchi, Junko Kagyo

**KINSHUKAI Hanwa The Second Hospital**

Minoru Takada, Hidenori Kanda

**Kiryu Kosei General Hospital**

Mitsuyoshi Utsugi, Akihiro Ono

**Kitasato University**

Tomomi Takano, Kazuhiko Katayama

**Kitasato University Kitasato Institute Hospital**

Yusuke Suzuki, Sohei Nakayama, Keita Masuzawa

**KKR Sapporo Medical Center**

Satoshi Fuke, Hiroshi Saito

**Kobe University**

Shohei Makino, Moritoki Egi

**Kumamoto City Hospital**

Hajime Iwagoe, Hiroshi Takahashi, Kazuhiko Fujii, Hiroto Kishi

**Kyoto Prefectural University of Medicine**

Satoru Hashimoto, Masaki Yamasaki, Yu Kasamatsu

**Kyoto University**

Ryunosuke Saiki, Yasuhito Nannya, Seishi Ogawa

**Kyushu University**

Satoru Fukuyama, Yoshihiro Eriguchi, Akiko Yonekawa, Keiko Kan-o, Koichiro Matsumoto

**Matsumoto City Hospital**

Akihiro Ito

**Musashino Red Cross Hospital**

Namiki Izumi, Kaoru Nagata, Ken Ueda, Reiko Taki, Satoko Hanada

**Nagoya University**

Naozumi Hashimoto, Keiko Wakahara, Sakamoto Koji, Norihito Omote, Akira Ando

**National Center for Global Health and Medicine**

Yosuke Omae, Katsushi Tokunaga

**National Defense Medical College**

Yoshifumi Kimizuka, Akihiko Kawana, Tomoya Sano, Chie Watanabe, Ryohei Suematsu

**National Hospital Organization Hokkaido Medical Center**

Toshio Odani, Masaru Amishima, Takeshi Hattori, Yasuo Shichinohe

**National Hospital Organization Kanazawa Medical Center**

Takashi Kagaya, Toshiyuki Kita, Kazuhide Ohta, Satoru Sakagami, Kiyoshi Koshida

**National Hospital Organization Kumamoto Medical Center**

Masahiro Harada, Takeshi Takahashi, Hiroshi Ono, Toshihiro Sakurai, Takayuki Shibusawa

**National Hospital Organization Kyoto Medical Center**

Kei Nishiyama, Mariko Terashima, Satoru Beppu, Kosuke Yoshida

**National Hospital Organization Kyushu Medical Center**

Masaki Okamoto, Sayoko Ishihara, Masatoshi Shimo, Yoshihisa Tokunaga

**National Hospital Organization Saitama Hospital**

Shinichi Hayashi, Mai Takahashi, Mizuki Kuramochi, Isamu Kamimaki, Yoshiteru Tominaga

**National Hospital Organization Tokyo Hospital**

Osamu Narumoto, Hideaki Nagai, Nobuharu Ooshima

**National Hospital Organization Tokyo Medical Center**

Takao Mochimaru, Shigenari Nukaga, Ryosuke Satomi, Yoshitaka Oyamada, Nobuaki Mori

**Nihon University Itabashi Hospital**

Yasuhiro Gon, Kentaro Hayashi, Tetsuo Shimizu, Yutaka Kozu, Hisato Hiranuma

**Nippon Medical School**

Toru Tanaka, Takeru Kashiwada, Kazue Fujita, Yoshinobu Saito, Masahiro Seike

**Okayama Rosai Hospital**

Arihiko Kanehiro, Shinji Ozaki, Yasuko Fuchimoto, Sae Wada, Nobukazu Fujimoto

**Ome Municipal General Hospital**

Yu Kusaka, Takehiko Ohba, Susumu Isogai, Aki Ogawa, Takuya Inoue

**Osaka Saiseikai Nakatsu Hospital**

Tetsuya Ueda, Masanori Azuma, Ryuichi Saito, Toshikatsu Sado, Yoshimune Miyazaki, Ryuichi Sato, Yuki Haruta, Tadao Nagasaki, Yoshinori Yasui, Yoshinori Hasegawa

**Osaka University**

Ryuya Edahiro, Yuya Shirai, Kyuto Sonehara, Daisuke Okuzaki, Daisuke Motooka, Masahiro Kanai, Tatsuhiko Naito, Kenichi Yamamoto, Qingbo S Wang, Yasuhiro Kato, Takayoshi Morita, Shinichi Namba, Ken Suzuki, Yoko Naito, Yu-Chen Liu, Ayako Takuwa, Fuminori Sugihara, James B Wing, Shuhei Sakakibara, Nobuyuki Hizawa, Takayuki Shiroyama, Satoru Miyawaki, Yusuke Kawamura, Akiyoshi Nakayama, Hirotaka Matsuo, Yuichi Maeda, Takuro Nii, Yoshimi Noda, Takayuki Niitsu, Yuichi Adachi, Takatoshi Enomoto, Saori Amiya, Reina Hara, Yuta Yamaguchi, Teruaki Murakami, Tomoki Kuge, Kinnosuke Matsumoto, Yuji Yamamoto, Makoto Yamamoto, Midori Yoneda, Toshihiro Kishikawa, Shuhei Yamada, Shuhei Kawabata, Noriyuki Kijima, Masatoshi Takagaki, Noah Sasa, Yuya Ueno, Motoyuki Suzuki, Norihiko Takemoto, Hirotaka Eguchi, Takahito Fukusumi, Takao Imai, Munehisa Fukushima, Haruhiko Kishima, Hidenori Inohara, Kazunori Tomono, Kazuto Kato, Meiko Takahashi, Fumihiko Matsuda, Haruhiko Hirata, Yoshito Takeda, Atsushi Kumanogoh, Yukinori Okada

**St. Marianna University School of Medicine**

Tomoya Tsuchida, Shigeki Fujitani, Mumon Takita, Daiki Morikawa, Toru Yoshida

**St. Marianna University School of Medicine, Yokohama-City Seibu Hospital**

Yuko Komase, Naoya Hida, Takahiro Tsuburai, Baku Oyama

**Saiseikai Kumamoto Hospital**

Kodai Kawamura, Kazuya Ichikado, Kenta Nishiyama, Hiroyuki Muranaka, Kazunori Nakamura

**Saiseikai Utsunomiya Hospital**

Ichiro Nakachi, Rie Baba, Daisuke Arai, Takayuki Ogura, Hidenori Takahashi, Shigehiro Hagiwara, Genta Nagao, Shunichiro Konishi

**Saiseikai Yokohamashi Nanbu Hospital**

Naoki Miyazawa, Yasuhiro Kimura, Reiko Sado, Hideyasu Sugimoto, Akane Kamiya

**Saitama Cardiovascular and Respiratory Center**

Takashi Ishiguro, Taisuke Isono, Shun Shibata, Yuma Matsui, Chiaki Hosoda, Kenji Takano, Takashi Nishida, Yoichi Kobayashi, Yotaro Takaku, Noboru Takayanagi

**Saitama City Hospital**

Hiroki Tateno, Isano Hase, Shuichi Yoshida, Shoji Suzuki, Miki Kawada, Hirohisa Horinouchi

**Sano Kosei General Hospital**

Takashi Inoue, Takahiro Asami, Toshiyuki Hirano, Keigo Kobayashi, Hatsuyo Takaoka

**Sapporo City General Hospital**

Hisako Sageshima

**Showa University**

Hironori Sagara, Akihiko Tanaka, Shin Ohta, Tomoyuki Kimura

**Showa University Koto Toyosu Hospital**

Naota Kuwahara, Akiko Fujiwara, Tomohiro Matsunaga, Yoko Sato, Takenori Okada

**Tachikawa Hospital**

Hidefumi Koh, Tadashi Manabe, Yohei Funatsu, Fumimaro Ito, Takahiro Fukui, Keisuke Shinozuka, Sumiko Kohashi, Masatoshi Miyazaki

**Toho University Ohashi Medical Center**

Hiroto Matsuse, Norio Kodaka, Chihiro Nakano, Takeshi Oshio, Takatomo Hirouchi

**Tohoku University**

Mitsuhiro Yamada, Koji Murakami, Hisatoshi Sugiura, Hirohito Sano, Shuichiro Matsumoto, Nozomu Kimura, Yoshinao Ono, Hiroaki Baba

**Tokai University**

Koichiro Asano, Tsuyoshi Oguma, Yoko Ito

**Tokyo Institute of Technology**

Takafumi Ueno

**Tokyo Medical and Dental University**

Ryuji Koike, Kunihiko Takahashi, Tatsuhiko Anzai, Satoshi Ito, Akifumi Endo, Yuji Uchimura, Yasunari Miyazaki, Takayuki Honda, Tomoya Tateishi, Shuji Tohda, Naoya Ichimura, Kazunari Sonobe, Chihiro Tani Sassa, Jun Nakajima, Masumi Ai, Akinori Kimura, Takanori Hasegawa, Satoru Miyano

**Tokyo Medical University Hospital**

Shinji Abe, Yuta Kono, Yuki Togashi, Hiroyuki Takoi, Ryota Kikuchi

**Tokyo Medical University Ibaraki Medical Center**

Tomoo Ishii

**Tokyo Metropolitan Police Hospital**

Masayuki Kanai, Tomonori Imamura, Tatsuya Yamashita

**Tokyo Saiseikai Central Hospital**

Ayumi Yoshifuji, Kazuto Ito, Saeko Takahashi, Kota Ishioka, Morio Nakamura

**Tokyo Women’s Medical University**

Etsuko Tagaya, Masatoshi Kawana, Ken Arimura

**Tokyo Women’s Medical University Medical Center East**

Tomohisa Shoko, Mitsuaki Kojima, Tomohiro Adachi, Motonao Ishikawa, Kenichiro Takahashi

**Tosei General Hospital**

Yoshikazu Mutoh, Tomoki Kimura, Tomonori Sato, Reoto Takei, Satoshi Hagimoto, Yoichiro Noguchi, Yasuhiko Yamano, Hajime Sasano, Sho Ota

**Toyohashi Municipal Hospital**

Tomoya Baba, Yasutaka Fukui, Mitsuru Odate, Shuko Mashimo, Yasushi Makino

**Tsukuba Kinen General Hospital**

Hiroko Watanabe

**Uji-Tokushukai Medical Center**

Yusuke Chihara, Mayumi Takeuchi, Keisuke Onoi, Jun Shinozuka, Atsushi Sueyoshi

**University of Tsukuba**

Yoshiaki Inoue, Shigeru Chiba, Kunihiro Yamagata, Yuji Hiramatsu, Hirayasu Kai

**Yamagata University**

Masafumi Watanabe, Sumito Inoue, Akira Igarashi, Masamichi Sato

**Yokohama City University**

Koji Okudela

**Yokohama Municipal Citizen’s Hospital**

Hiroyuki Hayashi, Yukihiro Yoshimura, Natsuo Tachikawa
